# Effectiveness of 23-Valent Pneumococcal Polysaccharide Vaccine against Invasive Pneumococcal Disease in Adults, Japan, 2013–2017

**DOI:** 10.3201/eid2610.191531

**Published:** 2020-10

**Authors:** Reiko Shimbashi, Motoi Suzuki, Bin Chang, Hiroshi Watanabe, Yoshinari Tanabe, Koji Kuronuma, Kengo Oshima, Takaya Maruyama, Hiroaki Takeda, Kei Kasahara, Jiro Fujita, Junichiro Nishi, Tetsuya Kubota, Keiko Tanaka-Taya, Tamano Matsui, Tomimasa Sunagawa, Kazunori Oishi, Adult IPD Study Group

**Affiliations:** National Institute of Infectious Diseases, Tokyo, Japan (R. Shimbashi, M. Suzuki, B. Chang, K. Tanaka-Taya, T. Matsui, T. Sunagawa, K. Oishi);; Tohoku University Graduate School of Medicine, Miyagi, Japan (R. Shimbashi, M. Suzuki, K. Oshima);; Kurume University School of Medicine, Fukuoka, Japan (H. Watanabe);; Niigata Prefectural Shibata Hospital, Niigata, Japan (Y. Tanabe);; Sapporo Medical University School of Medicine, Hokkaido, Japan (K. Kuronuma);; National Hospital Organization Mie National Hospital, Mie, Japan (T. Maruyama);; Yamagata Saisei Hospital, Yamagata, Japan (H. Takeda);; Nara Medical University, Nara, Japan (K. Kasahara);; Graduate School of Medicine, University of the Ryukyus, Okinawa, Japan (J. Fujita);; Kagoshima University Graduate School of Medical and Dental Sciences, Kagoshima, Japan (J. Nishi);; Kochi Medical School, Kochi University, Kochi, Japan (T. Kubota);; Toyama Institute of Health, Toyama, Japan (K. Oishi)

**Keywords:** PPSV23, 23-valent pneumococcal polysaccharide vaccine, IPD, invasive pneumococcal disease, vaccine effectiveness, indirect cohort method, indirect effect, respiratory diseases, vaccine-preventable diseases, Japan

## Abstract

The decline in the proportion of pneumococcal conjugate vaccine (PCV)–covered serotypes among adult invasive pneumococcal disease (IPD) patients might change the overall effectiveness of the 23-valent pneumococcal polysaccharide vaccine (PPSV23) because its effectiveness differs according to serotype. Using the indirect cohort method, we calculated the effectiveness of PPSV23 against IPD among adults in Japan to assess the impact of the national pediatric PCV program. Clinical and epidemiologic information and pneumococcal isolates were collected from IPD patients >20 years of age through enhanced IPD surveillance during April 2013–December 2017. Adjusted effectiveness against PPSV23-serotype IPD was 42.2%. Despite a substantial decline in the proportion of 13-valent PCV serotypes during the study period (45% to 31%), the change in effectiveness for PPSV23-serotype IPD was limited (47.1% to 39.3%) and only marginal in the elderly population (39.9% to 39.4%). The pediatric PCV program had limited impact on PPSV23 effectiveness against IPD in adults.

*Streptococcus pneumoniae* is a major cause of illness and death among adults (*1*). Pneumonia is the most common form of pneumococcal disease in adults, whereas invasive pneumococcal disease (IPD), including meningitis and bacteremia, has severe clinical manifestations with a high case-fatality ratio (*2*). Because incidence of adult IPD is high among older adults, it is a public health concern, particularly in countries with aging populations, such as Japan (*3*,*4*).

Two types of pneumococcal vaccines are currently available for adults: the 23-valent pneumococcal polysaccharide vaccine (PPSV23) and the 13-valent pneumococcal conjugate vaccine (PCV13). According to a systematic review, the effectiveness of PPSV23 against IPD among adults >50 years of age was 54% (*5*). The CAPITA trial showed that the efficacy of PCV13 against IPD among adults >65 years of age was 75% (*6*). Since 2014, both PPSV23 and PCV13 have been recommended for older persons in the United States (*7*), whereas only PPSV23 is recommended in other high-income countries.

Discussions over adult pneumococcal vaccination programs are complicated because of differences in vaccine effectiveness (VE) by serotype and age group. Previous studies have suggested that VE of PPSV23 differs by serotype (*8*,*9*), so overall VE might vary on the basis of the distribution of serotypes among the vaccinated population. In many countries, the proportion of PCV-covered serotypes among adult IPD patients has been decreasing since the introduction of pediatric PCVs (*10*–*13*); this decline might have changed the effectiveness of PPSV23 against PPSV23-serotype IPD. However, the effect of pediatric PCV programs on VE of PPSV23 among adults has not been fully established. In addition, a few studies have suggested that VE is lower among older adults, but evidence is limited (*9*,*14*).

In Japan, PCV7 was licensed in November 2010, included in the routine immunization program for children in April 2013, and replaced by PCV13 in October 2013; PPSV23 was included in the routine immunization program for adults >65 years of age in 2014. We conducted this study to investigate whether VE of PPSV23 against IPD among adults >20 years of age was affected by the pediatric PCV program. We assessed the change in the proportion of PCV-covered serotypes among adult IPD patients across the study period and that in overall VE. We also explored the differences in VE according to age group and other population characteristics.

## Methods

### Study Population

Under the revised Infectious Disease Control Law, national IPD surveillance was implemented in Japan in 2013. Since then, physicians have been required to report all IPD patients and their basic information to the nearest local public health centers. To collect more detailed clinical and epidemiologic information and *S. pneumoniae* isolates from adult IPD patients, in April 2013 the Adult IPD Study Group initiated enhanced surveillance covering 10 of the 47 prefectures of Japan (*15*). Details of the study design and methods have been described elsewhere (*15*,*16*). In brief, all IPD patients >15 years of age who had been identified in the local health centers were recruited for enhanced surveillance, and their clinical and epidemiologic information, including PPSV23 vaccination history, was collected by research collaborators by using a standardized case form. *S. pneumoniae* isolates and clinical specimens were collected from hospital laboratories or prefectural public health institutes and transferred to the National Institute of Infectious Diseases for further testing. A patient was defined as having IPD if the culture was positive for *S. pneumoniae* or if *S. pneumoniae*–specific DNA (*lytA* gene) was detected by PCR assay in samples collected from normally sterile sites, such as blood and cerebrospinal fluid. To investigate the effectiveness of PPSV23 among adults, we excluded IPD patients >15–19 years in this study. IPD patients >20 years of age who had been enrolled in the study during April 2013–December 2017 and whose clinical and epidemiologic information and microbiologic testing results were available were included in the analyses.

### Microbiological Testing

*S. pneumoniae* isolates were serotyped by using the capsule Quellung reaction with rabbit antisera (Statens Serum Institute, https://en.ssi.dk) after culturing overnight. Clinical specimens were serotyped by using the multiplex serotyping PCR assay as described previously (*15*,*17*). Because the Quellung reaction could not distinguish between serotypes 11A and 11E, these serotypes were grouped into serogroup 11A/E and considered to be the PPSV23 serotype. Isolates that did not react with any antiserum were classified as nontypeable. One isolate per patient was included in our analysis.

### Pneumococcal Vaccination Policy in Japan

In Japan, PPSV23 was included in the national immunization program in October 2014 for all persons >65 years of age and those 60–65 years of age with underlying diseases, such as heart disease, kidney disease, respiratory disease, and immunocompromised condition attributable to HIV infection. The cost of vaccination is partly subsidized by the local government. A national catch-up campaign targeting persons >65 years of age also was launched in 2014. For persons 2–59 years of age with high-risk conditions (e.g., a history of splenectomy), the cost of vaccination is covered by health insurance. According to an estimate by Japan’s Ministry of Health, Labor, and Welfare, 33%–38% of persons >65 years of age were vaccinated with PPSV23 during 2014–2017 (*18*). In our study, PPSV23 vaccination status was obtained from medical records and confirmed by patients or their guardians. Patients were considered vaccinated only if they had received >1 dose of PPSV23 in the 5 years before the hospital visit.

PCV7 was approved for the voluntary vaccination of children in February 2010, included in the routine immunization program for children in April 2013, and replaced with PCV13 in November 2013. The coverage rate of the third dose of PCV13 among children was 98% in 2017 (*18*). PCV13 was approved for adults >65 years of age in June 2014, but its coverage rate among this age group remains very low (<1%). In our study, only 4 of 1,121 patients reported having been vaccinated with PCV13.

### Procedures

VE of PPSV23 against IPD was calculated by using an indirect cohort method (Broome’s method). The indirect cohort method is a case-control design and has been used to estimate VE of pneumococcal vaccines by using pneumococcal disease surveillance data (*9*,*19*). In our study, a case was defined as illness in a patient with IPD caused by a PPSV23 serotype (PPSV23-serotype IPD), including serotypes 1, 2, 3, 4, 5, 6B, 7F, 8, 9N, 9V, 10A, 11A, 12F, 14, 15B, 17F, 18C, 19A, 19F, 20, 22F, 23F, and 33F; a control was defined as a patient with IPD caused by a non-PPSV23 serotypes. For the serotype-specific VE estimates, a case was defined as illness in patients with IPD caused by a specific serotype, and a control was defined as a patient with IPD caused by a non-PPSV23 serotype. We compared the odds of PPSV23 vaccination history in cases and controls and calculated VE as (1 – odds ratio) × 100%.

The patients were classified into 4 age groups: 20–39, 40–64, 65–79, and 80 years of age. Patients were considered to have immunocompromised conditions if they had any of the following conditions: a history of splenectomy, transplantation, asplenia or hyposplenia, HIV infection, malignancy, autoimmune disease, and complement deficiency (*20*,*21*). The patients’ body mass index (BMI) values were grouped as low (<18.5 kg/m^2^), normal (18.5–24.9 kg/m^2^), and high (>25 kg/m^2^). Clinical manifestations in IPD patients were classified as pneumonia, meningitis, bacteremia, and other conditions according to the physicians’ report. Because the IPD incidence is higher in the autumn and winter seasons compared with other seasons (*22*), we defined a high season (epidemiologic weeks 1–22 and 49–52) and a low season (epidemiologic weeks 23–48). To assess the effect of the pediatric PCV program, we divided the study period into 2 phases according to the year of diagnosis: the first phase (2013–2015) and the second phase (2016–2017).

### Statistical Analyses

The characteristics of cases and controls were compared by using the χ^2^ test or Fisher exact test, as appropriate. We used logistic regression models to estimate VE. Because sex, age, study site, year of diagnosis, season, BMI group, presence of an underlying condition, and smoking status were deemed to be potential confounders on the basis of prior knowledge (*9*), all factors were included in the final multiple logistic regression models. CIs were adjusted for clustering at the local health center level by using robust SEs.

We estimated VE of IPD for PPSV23 serotypes, PCV13 serotypes excluding 6A (PCV13 non–6A serotype), including serotypes 1, 3, 4, 5, 6B, 7F, 9V, 14, 18C, 19A, 19F, and 23F, and PPSV23 serotypes excluding PCV13 serotypes (PPSV23 non–PCV13 serotypes). The serotype-specific VE was estimated for each serotype if its number of isolates was >30. To explore the potential effect of cross-immunity produced by PPSV23 on serogroup 6 (*23*), VE of IPD for serotypes 6A, 6B, 6C, and 6D was calculated excluding these serotypes from the controls. VE was stratified by the 2 study phases (2013–2015 and 2016–2017). We also conducted stratified analyses to investigate the potential effect of modifications by sex, age group (persons <65 years of age and those >65 years of age), the presence of underlying conditions, BMI group, and clinical manifestations. The stratum-specific estimates of VE were compared by using a Wald test (test for interaction).

PPSV23 vaccination history was not documented for 23% of our patients. This group was coded as no record and included in our primary analyses. In a sensitivity analysis, this group was considered to have missing data, and multiple imputations were performed. All analyses were performed by using Stata version 15 (StataCorp, https://www.stata.com).

### Ethics

This study was approved by the Ethics Committee of the National Institute of Infectious Diseases (approval no. 707) and conducted according to the principles expressed in the Declaration of Helsinki. The requirement for obtaining informed consent from all participants was waived because the data do not contain any patient identifiers, and samples were taken as part of standard patient care.

## Results

During the study period (April 2013–December 2017), a total of 1,824 IPD patients >20 years of age were identified through the national IPD surveillance program in the study prefectures (*24*). Among them, 1,138 patients were enrolled in the study (Appendix Figure). *S. pneumoniae* isolates or clinical specimens were not available for 15 patients, and clinical and epidemiologic data were not available for an additional 2 patients. After excluding these patients, 1,121 patients were eligible for our analyses. *S. pneumoniae* was identified in 1,117 patients (99.6%), and *S. pneumoniae*–specific DNA was detected in 4 patients (0.4%).

The characteristics of the 1,121 IPD patients are summarized in Table 1. A total of 679 (61%) patients were men, and the median age was 70 years (range 22–103 years). Among all IPD patients, 746 (66.5%) were classified as having PPSV23-serotype IPD and 375 (33.5%) were classified as having non–PPSV23-serotype IPD. PPSV23-serotype IPD patients less frequently had immunocompromised conditions and more frequently had pneumonia compared with non–PPSV23-serotype IPD patients; otherwise, characteristics were similar between the 2 groups.

**Table 1:** Characteristics of 1,121 invasive pneumococcal disease patients with and without PPSV23 serotype, Japan, 2013–2017*

Characteristic	Total		PPSV23 serotype, n = 746		Non-PPSV23 serotype, n = 375		p value
Sex							
M	679 (61)		443 (59)		236 (63)		0.251
F	442 (39)		303 (41)		139 (37)		
Age group, y							
20–39	55 (5)		34 (5)		21 (6)		0.437
40–64	309 (28)		211 (28)		98 (26)		
65–79	427 (38)		291 (39)		136 (36)		
>80	330 (29)		210 (28)		120 (32		
Study site, prefecture							
Hokkaido	138 (12)		85 (11)		53 (14)		0.612
Miyagi	133 (12)		92 (12)		41 (11)		
Yamagata	95 (8)		69 (9)		26 (7)		
Niigata	211 (19)		144 (19)		67 (18)		
Mie	113 (10)		78 (10)		35 (9)		
Nara	80 (7)		51 (7)		29 (8)		
Kochi	38 (3)		27 (4)		11 (3)		
Fukuoka	222 (20)		146 (20)		76 (20)		
Kagoshima	45 (4)		28 (4)		17 (5)		
Okinawa	46 (4)		26 (3)		20 (5)		
Year							
2013	45 (4)		33 (4)		12 (3)		0.602
2014	201 (18)		134 (18)		67 (18)		
2015	213 (19)		146 (20)		67 (18)		
2016	286 (26)		180 (24)		106 (28)		
2017	363 (32)		243 (33)		120 (32)		
Unknown	13 (1)		10 (1)		3 (1)		
Season†							
High season	722 (64)		492 (66)		230 (61)		0.152
Low season	385 (34)		243 (33)		142 (38)		
Unknown	14 (1)		11 (1)		3 (1)		
BMI group, kg/m^2^							
<18.5	257 (23)		171 (23)		86 (23)		0.895
18.5–24.9	526 (47)		346 (46)		180 (48)		
>25	167 (15)		111 (15)		56 (15)		
Unknown	171 (15)		118 (16)		53 (14)		
Underlying conditions							
Immunocompromised conditions	314 (28)		175 (23)		139 (37)		<0.001
Other conditions	479 (43)		324 (43)		155 (41)		
Without underlying conditions	256 (23)		198 (27)		58 (15)		
Unknown	72 (6)		49 (7)		23 (6)		
Smoking history							
Yes	390 (35)		265 (36)		125 (33)		0.709
No	559 (50)		370 (50)		189 (50)		
Unknown	172 (15)		111 (15)		61 (16)		
Alcohol intake							
Yes	184 (16)		121 (16)		63 (17)		0.439
No	750 (67)		493 (66)		257 (69)		
Unknown	187 (17)		132 (18)		55 (15)		
Clinical manifestations							
Pneumonia	665 (59)		480 (64)		185 (49)		<0.001
Meningitis	169 (15)		94 (13)		75 (20)		
Bacteremia	188 (17)		104 (14)		84 (22)		
Other‡	98 (9)		68 (9)		30 (8)		
Unknown	1 (0)		0 (0)		1 (0)		
Fatal outcome							
Yes	204 (18)		137 (18)		67 (18)		0.838
No	917 (82)		609 (82)		308 (82)		
PPSV23 vaccination within 5 y							
Yes	103 (9)		58 (8)		45 (12)		<0.001
No	765 (68)		539 (72)		226 (60)		
Unknown	253 (23)		149 (20)		104 (28)		

*Values are no. (%) unless indicated. BMI, body mass index; EW, epidemiologic week; PPSV23, 23-valent pneumococcal polysaccharide vaccine. †High season indicates the period from EW 1 to EW 22 and from EW 49 to EW 52, whereas low season indicates the period from EW 23 to EW 48. ‡Includes arthritis, endocarditis, sinusitis, otitis media, vertebritis, cholecystitis, aortic aneurysm, and pleurisy.

After we controlled for confounders, VE against PPSV23-serotype IPD was 42.2% (95% CI 13.4 to 61.4) (Table 2). VE against PCV13 non–6A-serotype IPD was 35.3% (95% CI −8.4% to 61.5%) and that against PPSV23 non–PCV13-serotype IPD was 44.5% (95% CI 9.6% to 65.9%). Sensitivity analyses showed similar VE estimates (Appendix Table 1). A high level of effectiveness was observed for serotypes 19A (70.3% [95% CI 13.3% to 89.8%]), 12F (70.8% [95% CI 1.0% to 91.4%]), and 10A (73.6% [95% CI 5.9% to 92.6%]). Low-to-moderate effectiveness was observed for serotypes 3 (34.1% [95% CI −34.4% to 67.7%]), 22F (22.7% [95% CI −88.8% to 68.4%]), 11A/E (20.7% [95% CI −145.4% to 74.4%]), and 7F (22.4% [95% CI −176.8% to 78.2%]); however, their CIs were wide because of the limited sample size.

**Table 2:** Overall and serotype-specific effectiveness of PPSV23 against invasive pneumococcal disease in adults >20 years of age, Japan, 2013–2017*

Serotype	No. cases	No. controls	Crude VE, % (95% CI)	Adjusted VE,† % (95% CI)
PPSV23 serotype	746	375	46.0 (17.8 to 64.5)	42.2 (13.4 to 61.4)
PCV13, non–6A serotype	392	375	40.6 (3.8 to 63.3)	35.3 (−8.4 to 61.5)
PPSV23, non–PCV13 serotype	354	375	51.7 (18.7 to 71.3)	44.5 (9.6 to 65.9)
Serotype 3	152	375	43.1 (−11.9 to 71.1)	34.1 (−34.4 to 67.7)
Serotype 19A	111	375	72.7 (29.1 to 89.5)	70.3 (13.3 to 89.8)
Serotype 12F	99	375	80.2 (34.4 to 94.0)	70.8 (1.0 to 91.4)
Serotype 22F	83	375	34.1 (−47.1 to 70.5)	22.7 (−88.8 to 68.4)
Serotype 10A	80	375	75.7 (19.2 to 92.7)	73.6 (5.9 to 92.6)
Serotype 11A/E	41	375	30.7 (−106.7 to 76.8)	20.7 (−145.4 to 74.4)
Serotype 7F	30	375	31.5 (−138.6 to 80.3)	22.4 (−176.8 to 78.2)

*BMI, body mass index; PCV13, 13-valent pneumococcal conjugate vaccine; PPSV23, 23-valent pneumococcal polysaccharide vaccine; VE, vaccine effectiveness. †Adjusted for sex, age, prefecture, year, season, BMI group, underlying conditions, and smoking history with clustering by public health center.

The trend in the proportion of vaccine-covered serotypes among adult IPD patients during the study period is shown in the Figure. The proportion of PCV13 serotypes was 45% in the first phase of the study (2013–2015) and 31% in the second phase (2016–2017). When we stratified the patients by age group, the decline was 24% (41% in the first phase and 17% in the second phase) among patients 20–64 years of age and 10% (47% in the first phase and 37% in the second phase) among those >65 years of age.

**Figure:**
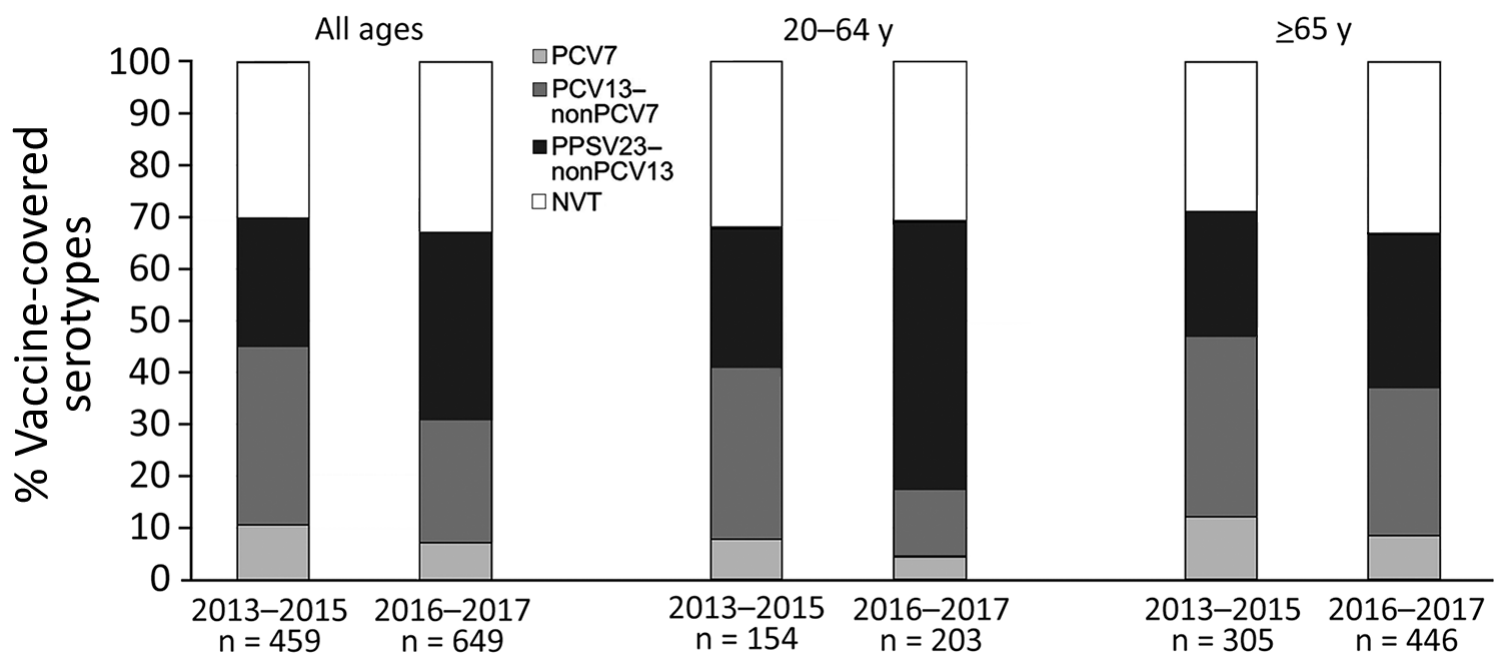
Percentage of vaccine-covered serotypes among pneumococcal isolates from 1,108 invasive pneumococcal disease patients >20 years of age, stratified by year and age group, Japan, 2013–2017. NVT, non–vaccine type; PCV7, 7-valent pneumococcal conjugate vaccine; PCV13, 13-valent pneumococcal conjugate vaccine; PPSV23, 23-valent pneumococcal polysaccharide vaccine.

VE against PPSV23-serotype IPD among persons >20 years of age in the first phase was 47.1% (95% CI −4.7% to 73.3%) and in the second phase was 39.3% (95% CI −2.9% to 64.2%) (p = 0.953 by test for interaction) (Table 3). When we focused on persons >65 years of age, VE point estimates in the 2 phases showed almost identical values (39.9% in the first phase and 39.4% in the second; p = 0.809). For persons 20–64 years of age, VE point estimates showed a decreasing trend (77.1%in the first phase and 41.0% in the second; p = 0.124); however, the CIs were wide.

**Table 3:** Effectiveness of PPSV23 against invasive pneumococcal disease in adults >20 years of age, by age group and study period, Japan, 2013–2017*

Age group, y	Adjusted VE,† % (95% CI)		p value by test for interaction
	2013–2015	2016–2017	
Overall	47.1 (−4.7 to 73.3)	39.3 (−2.9 to 64.2)	0.953
20–64	77.1 (−110.4 to 97.5)	41.0 (−128.8 to 84.8)	0.124
>65	39.9 (−28.4 to 71.9)	39.4 (−6.1 to 65.3)	0.809

*BMI, body mass index; PPSV23, 23-valent pneumococcal polysaccharide vaccine; VE, vaccine effectiveness. †Adjusted for sex, age, prefecture, year, season, BMI group, underlying conditions, and smoking history with clustering by public health center.

Our subgroup analyses showed that VE against PPSV23-serotype IPD was 59.0% (95% CI 17.9% to 79.6%) among persons 20–64 years of age and 39.2% (95% CI 2.0% to 62.2%) among those >65 years of age, but this difference was not statistically significant level (p = 0.17 by test for interaction) (Table 4). When we stratified the age group further, VE was 44.6% (95% CI −14.5% to 73.2%) among persons 65–79 years of age and 31.3% (95% CI −47.7% to 68.1%) among those >80 years of age. Higher VE was observed in persons with a normal BMI (70.6% [95% CI 47.7% to 83.5%]) than in those with a low BMI (7.4% [95% CI −108.1% to 58.8%]) or a high BMI (−136.5% [95% CI −826.6% to 39.6%]). VE did not differ by patients’ underlying diseases. Among persons >65 years of age, VE for IPD with pneumonia was 52.8% (95% CI 16.5% to 73.3%) and for IPD without pneumonia was 9.8% (95% CI −134.4% to 65.3%) (Appendix Table 2). Among persons 20–64 years of age, VE for IPD with pneumonia was 23.0% (95% CI −272.2% to 84.1%) and for IPD without pneumonia was 88.8% (95% CI −12.0% to 98.9%).

**Table 4:** Stratified analyses of the effectiveness of PPSV23 against invasive pneumococcal disease in adults >20 years of age, Japan, 2013–2017*

Characteristic	No. cases	No. controls	Crude VE, % (95% CI)	Adjusted VE,† % (95% CI)	p value
Sex					
M	443	236	41.5 (2.0 to 65.0)	38.7 (−5.1 to 64.3)	0.917	
F	303	139	52.7 (2.5 to 77.1)	48.5 (5.3 to 72.0)	
Age group, y					
20–64	245	119	72.5 (13.6 to 91.3)	59.0 (17.9 to 79.6)	0.170	
>65	501	256	41.7 (7.2 to 63.3)	39.2 (2.0 to 62.2)	
Clinical manifestations					
Pneumonia	480	185	55.8 (26.2 to 73.5)	50.6 (16.0 to 70.9)	0.284
Meningitis	94	75	46.9 (−76.6 to 84.1)	35.6 (−100.0 to 79.2)		
Bacteremia	104	84	41.8 (−78.2 to 81.0)	34.7 (−72.1 to 75.2)		
Other‡	68	30	NA	NA	
BMI group, kg/m^2^					
<18.5	171	86	11.3 (−109.4 to 62.4)	7.4 (−108.1 to 58.8)	0.005	
18.5–24.9	346	180	73.2 (50.8 to 85.4)	70.6 (47.7 to 83.5)		
>25	111	56	−133.3 (−765.0 to 37.1)	−136.5 (−826.6 to 39.6)	
Underlying conditions					
Immunocompromised	175	139	41.0 (−22.0 to 71.4)	41.2 (−27.6 to 72.9)	0.971	
Other condition	324	155	48.5 (7.2 to 71.4)	48.2 (6.0 to 71.5)		
No underlying condition	198	58	48.8 (−113.3 to 87.7)	51.5 (−116.0 to 89.1)	

*BMI, body mass index; NA, not available; PPSV23, 23-valent pneumococcal polysaccharide vaccine; VE, vaccine effectiveness. †Adjusted for sex, age, prefecture, year, season, BMI group, underlying conditions, and smoking history with clustering by public health center. ‡Includes arthritis, endocarditis, sinusitis, otitis media, vertebritis, cholecystitis, aortic aneurysm, and pleurisy.

## Discussion

VE of PPSV23 against PPSV23-serotype IPD was 42.2% among adults >20 years of age in Japan. VE against PCV13 non–6A-serotype IPD and that against PPSV23 non–PCV13-serotype IPD were almost comparable. Despite an observed reduction in the proportion of PCV13 serotypes among adult IPD patients during the study period, the change in VE against PPSV23-serotype IPD was limited among adults >20 years of age and only marginal among those >65 years of age.

Large declines in the incidence of adult pneumococcal disease caused by PCV serotypes have been reported in many countries because of the indirect effect of pediatric PCVs (*10*,*25*–*27*). A pooled analysis of 10 countries in Europe demonstrated that during 2009–2015, incidence of PCV7-serotype IPD among adults >65 years of age declined by 77% and incidence of PCV13 non–PCV7-serotype IPD among the same age group declined 38% (*26*). The indirect effect of pediatric PCVs is particularly important when making adult pneumococcal vaccination policies because it might affect the effectiveness and population impact of adult vaccines. In our study, the proportion of PCV13 serotypes among adult IPD patients decreased from 45% to 31%, whereas the proportion of PPSV23 non–PCV13 serotypes increased from 25% to 36%. Because the effectiveness of pneumococcal vaccines is known to differ by serotype, this change in the serotype distribution might have changed overall PPSV23 effectiveness. However, in our study, VE values against PCV13 non–6A-serotype IPD (35.3% [95% CI −8.4% to 61.5%]) and PPSV23 non–PCV13-serotype IPD (44.5% [95% CI 9.6% to 65.9%]) did not differ substantially. Consequently, the change in VE point estimates among adults >20 years of age was limited during the study period (47.1% in the first phase and 39.3% in the second), and no change was observed among those >65 years of age (39.9% in the first phase and 39.4% in the second). Our findings suggest that VE of PPSV23 among adults in Japan is moderate and remained constant during the 4-year study period under the impact of pediatric PCV13.

In the current study, VE values against IPD varied by serotype; high VE was observed against serotypes 19A, 12F, and 10A, and low-to-moderate VE was observed against serotypes 3, 22F, 11A/E, and 7F. Serotype 3 was the leading serotype observed in our patients, as has been the case in other countries (*28*). Studies have shown that the efficacy of pneumococcal vaccines against serotype 3 is limited (*28*). The observed low-to-moderate VE in our study (34.1% [95% CI −34.4% to 67.7%]) was consistent with previous studies (*8*,*9*,*29*). Recently, we reported the emergence of serotype 12F among adult IPD patients in Japan (*16*), and that serotype was the third leading serotype (9%) identified in our patients. Increases in incidence of serotype 12F have been observed in other countries after the introduction of pediatric PCVs (*30*,*31*). Studies have suggested that this serotype is associated with outbreaks and a high invasiveness potential (*32*–*34*). The high VE against serotype 12F (70.8% [95% CI 1.0% to 91.4%]) observed in our study suggests that PPSV23 vaccination is an effective measure to reduce its impact.

When we stratified the patients by age group, VE was 59% (95% CI 17.9% to 79.6%) for persons 20–64 years of age and 39.2% (95% CI 2% to 62.2%) for those >65 years of age; however, this difference was not statistically significant (p = 0.17 by test for interaction). A declining trend in PPSV23 effectiveness with age has been reported previously (*9*,*14*). A study conducted in Spain showed that VE against IPD was 54.2% in adults 60–69 years of age, 54.1% in adults 70–79 years of age, and 25.5% in adults >80 years of age (*9*). These observations might be explained by the decline in pneumococcal polysaccharide immunity with increasing age (*35*). We found that VE was lower among persons with a low or high BMI than among those with a normal BMI (p = 0.005 by test for interaction). Malnutrition, including undernutrition and overnutrition, is known to be associated with immune defects (*36*) and poor vaccine-induced immune responses (*37*). On the other hand, VE did not differ between persons with and without underlying conditions. A similar finding was observed in a previous study conducted in Japan; the effectiveness of PPSV23 against pneumococcal pneumonia among adults >65 years of age did not differ by their underlying condition status (*29*). These observations might be at least partially explained by the low prevalence of HIV infection in adults in Japan. Only 1 patient was recorded as being HIV-positive in our study.

VE for bacteremic pneumococcal pneumonia among persons >65 years of age was 52.8% (95% CI 16.5% to 73.3%), whereas among persons 20–64 years of age it was 23.0% (95% CI −272.2% to 84.1%) (p = 0.064 by test for interaction). Because pneumonia is the most common manifestation of pneumococcal disease among the elderly population (*15*), this finding might support the current PPSV23 recommendations. On the other hand, although the CI was wide, the VE point estimate for non–pneumonia-associated IPD, such as meningitis and occult bacteremia (i.e., bacteremia without an identifiable focus of infection), was high in the younger age group. The potential difference in PPSV23 effectiveness according to population characteristics and clinical manifestations is particularly important when creating efficient vaccination policies. Further studies are needed to understand the mechanisms underlying our observations.

In Japan, PPSV23 was introduced into the adult immunization program in 2014, but its vaccination coverage rate was only »30% in 2017. Recently, the Ministry of Health, Labor, and Welfare decided to extend the duration of the catch-up campaign for persons >65 years of age until 2023. The continued moderate PPSV23 effectiveness under the impact of the pediatric PCV13 program we observed provides supporting evidence for the current adult pneumococcal vaccination policy. However, making decisions regarding the adult PPSV23 program is still challenging for several reasons, such as its low level of efficacy in the older age group (*8*,*9*) and limited evidence supporting repeated vaccinations (*38*,*39*). Continuous monitoring of the serotype distribution and VE among adults is warranted. On the other hand, high VE among younger adults, particularly for meningitis and occult bacteremia, might facilitate a discussion regarding the potential expansion of the target age group.

Our study has limitations. First, 37.6% of the patients identified in the local health centers were not included in our study. The inclusion rate was especially low at the beginning of the study period; however, the baseline characteristics of the patients did not differ between the enrolled and nonenrolled patients (Appendix Table 3). The effect of selection bias on our VE estimates must have been minimal. Second, vaccination history was not documented in 23% of our patients. Our sensitivity analyses showed almost identical estimates, so we do not believe this shortcoming affects our observations. Third, we used the indirect cohort method, which is equivalent to the test-negative design, to estimate the PPSV23 effectiveness. Although this design is less susceptible to bias associated with confounding by healthcare-seeking behavior, as in the nature of observational study design, the bias is unlikely to be eliminated (*40*,*41*). However, our VE estimates are comparable with previous estimates resulting from other study designs (*5*), so the effect of bias is probably minimal. Finally, only a history of PPSV23 vaccination within 5 years was available. Patients who had received the latest PPSV23 >5 years before diagnosis were classified as unvaccinated. If VE lasted >5 years, our VE estimates might have underestimated actual VEs. Also, our study could not assess the waning of effectiveness over the 5 years.

In conclusion, the effectiveness of PPSV23 against IPD is moderate among adults >20 years of age in Japan. Although the proportion of PCV13 serotypes among adult IPD patients has been substantially decreasing because of the indirect effect of the pediatric PCV program, the change in PPSV23 effectiveness was limited.

Appendix Additional information about effectiveness of 23-valent pneumococcal polysaccharide vaccine against invasive pneumococcal disease in adults, Japan, 2013–2017.
