# Supplementary material for: Effectiveness of 23-Valent Pneumococcal Polysaccharide Vaccine against Invasive Pneumococcal Disease in Adults, Japan, 2013–2017
Source: Emerg Infect Dis. 2020 Oct;26(10):2378–86. doi: 10.3201/eid2610.191531 (PMC7510723; doi:10.3201/eid2610.191531)
Supplement: Appendix — Additional information about effectiveness of 23-valent pneumococcal polysaccharide vaccine against invasive pneumococcal disease in adults, Japan, 2013–2017. [file 19-1531-Techapp-s1.pdf]

Article DOI: https://doi.org/10.3201/eid2610.191531

Effectiveness of 23-Valent Pneumococcal Polysaccharide Vaccine against Invasive Pneumococcal Disease in Adults, Japan, 2013–2017

Appendix

Appendix Table 1. Sensitivity analyses of the effectiveness of PPSV23 against invasive pneumococcal disease in adults ≥20 years of age, Japan, 2013–2017*

Serogroup	No. cases	No. controls	Crude VE, % (95% CI)	Adjusted VE,† % (95% CI)
Overall VE using multiple imputation for missing vaccination history	746	375	45.9 (19.4 to 63.7)	43.5 (14.0 to 62.8)
Overall VE excluding serotypes 6A, 6C, and 6D from controls	746	293	48.8 (20.0 to 67.2)	46.0 (13.9 to 66.1)
VE for serogroup 6 (serotypes 6A, 6B, 6C and 6D)	108	293	22.0 (–62.0 to 62.3)	26.8 (–78.6 to 70.1)
VE for serotype 6A	20	355	62.7 (–192.1 to 95.2)	79.3 (–93.2 to 97.8)
VE for serotype 6B	26	375	11.3 (–215.1 to 75.0)	21.6 (–197.2 to 79.3)
VE for serotype 6C	59	316	–1.0 (–143.0 to 58.4)	–7.1 (–217.4 to 63.8)
VE for serotype 6D	3	372	NA	NA

*BMI, body mass index; NA, not available; PPSV23, 23-valent pneumococcal polysaccharide vaccine; VE, vaccine effectiveness.
†Adjusted for sex, age, prefecture, year, season, BMI group, underlying conditions, and smoking history with clustering by public health center.

Appendix Table 2. Effectiveness of PPSV23 against invasive pneumococcal disease in adults ≥20 years of age, by clinical manifestations and age group, Japan, 2013–2017*

Age group and pneumonia status	No. cases	No. controls	Crude VE, % (95% CI)	Adjusted VE,† % (95% CI)	p-value
20–64 y					
With pneumonia	122	36	47.1 (–204.5 to 90.8)	23.0 (–272.2 to 84.1)	0.064
Without pneumonia	123	83	89.6 (11.7 to 98.8)	88.8 (–12.0 to 98.9)	
≥65 y					
With pneumonia	358	149	55.0 (22.3 to 73.9)	52.8 (16.5 to 73.3)	0.390
Without pneumonia	143	106	11.6 (–122.1 to 64.8)	9.8 (–134.4 to 65.3)	

*BMI, body mass index; NA, not available; PPSV23, 23-valent pneumococcal polysaccharide vaccine; VE, vaccine effectiveness.
†Adjusted for sex, age, prefecture, year, season, BMI group, underlying conditions, and smoking history with clustering by public health center.

Appendix Table 3. Characteristics of invasive pneumococcal disease patients ≥20 years of age included and not included in enhanced surveillance, Japan, 2013–2017*

Characteristic	Patients included in enhanced surveillance, n = 1,138	Patients not included in enhanced surveillance, n = 686	p value
Sex			
Male	690 (61)	399 (58)	0.344
Female	446 (39)	287 (42)	
Unknown	2 (0)	0 (0)	
Age group, y			
20–39	56 (5)	38 (6)	0.217
40–64	313 (28)	174 (25)	
65–79	433 (38)	242 (35)	
≥80	334 (29)	232 (34)	
Unknown	2 (0)	0 (0)	
Year			
2013	45 (4)	85 (12)	<0.001
2014	206 (18)	101 (15)	
2015	216 (19)	159 (23)	
2016	288 (25)	158 (23)	
2017	366 (32)	183 (27)	
Unknown	17 (1)	0 (0)	

*Values are no. (%) unless indicated.

Appendix Table 4. Characteristics of invasive pneumococcal disease patients with known and unknown vaccination status, Japan, 2013–2017*

Characteristic	Patients with known vaccination history, n = 868	Patients with unknown vaccination history, n = 253	p value
Sex			
Male	536 (62)	143 (57)	0.134
Female	332 (38)	110 (43)	
Age group, y			
20–39	47 (5)	8 (3)	<0.001
40–64	254 (29)	55 (22)	
65–79	341 (39)	86 (34)	
≥80	226 (26)	104 (41)	
Year			
2013	37 (4)	8 (3)	<0.001
2014	170 (20)	31 (12)	
2015	177 (20)	36 (14)	
2016	220 (25)	66 (26)	
2017	261 (30)	102 (40)	
Unknown	3 (0)	10 (4)	

*Values are no. (%) unless indicated.

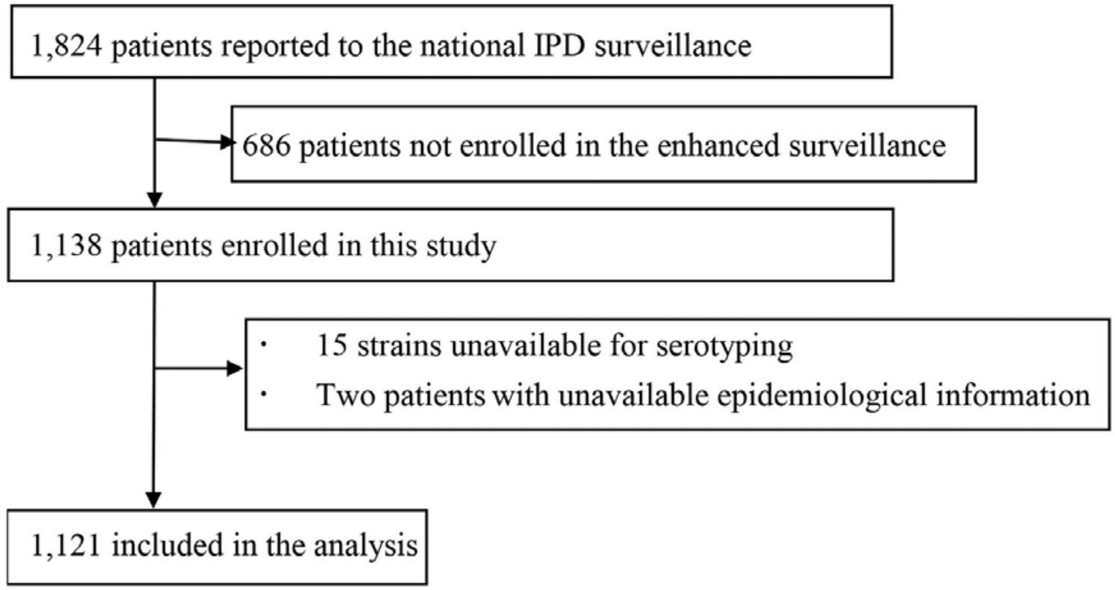

Appendix Figure. Study flow diagram.
